# Effect of topical dexamethasone and bupivacaine application on postoperative pain following total extraperitoneal hernioplasty: a randomized controlled prospective study

**DOI:** 10.1186/s12893-026-03627-3

**Published:** 2026-02-24

**Authors:** Gürkan Değirmencioğlu, Deniz Kütük, Mehmet Hanifi Çanakcı

**Affiliations:** Etlik City Hospital, Department of General Surgery, Ankara, Turkey

**Keywords:** Total Extraperitoneal Hernioplasty, Postoperative Pain, Dexamethasone, Bupivacaine

## Abstract

**Background:**

Although pain after laparoscopic total extraperitoneal (TEP) hernioplasty is lower compared with open techniques, effective pain control in the early postoperative period still remains clinically important. Although local anaesthetics are widely used, their limited duration of action often necessitates additional analgesics. This study was conducted to evaluate the effectiveness of the combination of dexamethasone and bupivacaine, applied locally to the surgical site, in reducing early postoperative pain and the need for analgesics.

**Methods:**

In this prospective, randomized controlled study, 175 patients who underwent elective unilateral TEP hernioplasty were allocated to the intervention (*n* = 87) and control (*n* = 88) groups. In the intervention group, after mesh placement, a combination of 4 mL 0.5% bupivacaine (20 mg) and 8 mg dexamethasone was topically applied to the surgical area via standard laparoscopic irrigation. No local pharmacological application was performed in the control group. In both groups, pain levels were assessed at the 3rd, 6th, and 12th postoperative hours using the Visual Analog Scale (VAS), along with additional analgesic requirements and patient satisfaction levels. The results were statistically compared between the groups.

**Results:**

VAS scores at the 3rd, 6th, and 12th hours were significantly lower in the intervention group receiving topical dexamethasone and bupivacaine compared to the control group (*p* < 0.001). The change in VAS scores was also significantly greater in the intervention group compared to the control group (3rd–6th hour: *p* = 0.005; 6th–12th hour: *p* < 0.001; 3rd–12th hour: *p* < 0.001). The proportion of patients requiring additional analgesics was significantly lower in the intervention group than in the control group (85.1% vs. 95.5%) (*p* = 0.020). Patient satisfaction scores were also significantly higher in the intervention group (*p* = 0.001).

**Conclusion:**

Local application of dexamethasone in combination with bupivacaine during TEP hernioplasty significantly reduces early postoperative pain and the need for additional analgesia while increasing patient satisfaction. This technique could be considered in laparoscopic hernia repair.

**Trial registration:**

This trial was retrospectively registered at ClinicalTrials.gov on October 8, 2025 (registration number NCT07208253).

## Introduction

Total extraperitoneal (TEP) hernioplasty is a widely used minimally invasive technique for inguinal hernia repair, known for shortening postoperative recovery time and for being associated with low complication rates [1–4]. However, following the TEP procedure, patients may experience significant postoperative pain due to factors such as tissue tension within the limited surgical field and nerve irritation that can cause pain [5]. Pain control is critical for early mobilization, initiation of oral intake, and shortening the length of hospital stay [6].

Regional applications of local anaesthetics for reducing postoperative pain have been frequently investigated in laparoscopic procedures such as TEP. Bupivacaine, one of the long-acting local anaesthetics, has been reported to reduce pain severity and the need for analgesics when applied to the extraperitoneal space [7, 8]. Some studies have shown that extraperitoneal instillation of bupivacaine significantly reduces pain and improves patient satisfaction [5, 6], whereas other investigations have reported that this effect is limited [7, 9].

In addition to bupivacaine, dexamethasone, a glucocorticoid known for its strong anti-inflammatory properties, has also been recognized as an effective agent in controlling postoperative pain. Dexamethasone exerts its analgesic effect by reducing neuronal transmission in peripheral nerves and suppressing the release of proinflammatory cytokines [10, 11]. The role of dexamethasone as an adjuvant to local anesthetics has been supported by randomized clinical trials and comprehensive meta-analyses demonstrating reduced postoperative pain and analgesic consumption with its use in regional anesthesia or wound infiltration techniques [12–14]. It has been reported that systemic or local administration of dexamethasone in patients undergoing laparoscopic surgery reduces analgesic consumption and significantly alleviates pain [11, 12].

Therefore, within the framework of multimodal approaches to controlling postoperative pain, the combined use of local anaesthetics and steroids has attracted increasing attention [12–14]. However, the number of randomized controlled trials evaluating the combined effect of bupivacaine and dexamethasone following the TEP procedure is limited [4, 15]. The aim of this study was to evaluate the effect of the combination of bupivacaine and dexamethasone on postoperative pain following TEP hernioplasty and to determine the potential contributions of this method to analgesic requirements and patient comfort.

## Materials and methods

### Study design

This study was designed as a single-center, prospective, randomized, controlled trial. It was conducted at the General Surgery Clinic of Kırıkhan State Hospital between April 2021 and December 2022 in patients undergoing planned TEP hernioplasty. The study protocol was carried out in accordance with the Declaration of Helsinki, and approval was obtained from the Clinical Research Ethics Committee of Mustafa Kemal University Tayfur Ata Sökmen Faculty of Medicine (Decision No: 2021/26, dated: 25.03.2021).

### Participant selection

Patients aged 18 years and older, with an ASA (American Society of Anaesthesiologists) score of I–II, who were scheduled for elective laparoscopic TEP inguinal hernia repair, and who provided written informed consent were included in the study. Conversely, patients with bilateral or recurrent inguinal hernia, a history of previous lower abdominal surgery, a history of analgesic or steroid use, contraindications to local or general anesthesia, those under immunosuppressive therapy, or with a history of malignancy were excluded.

**Fig. 1:**
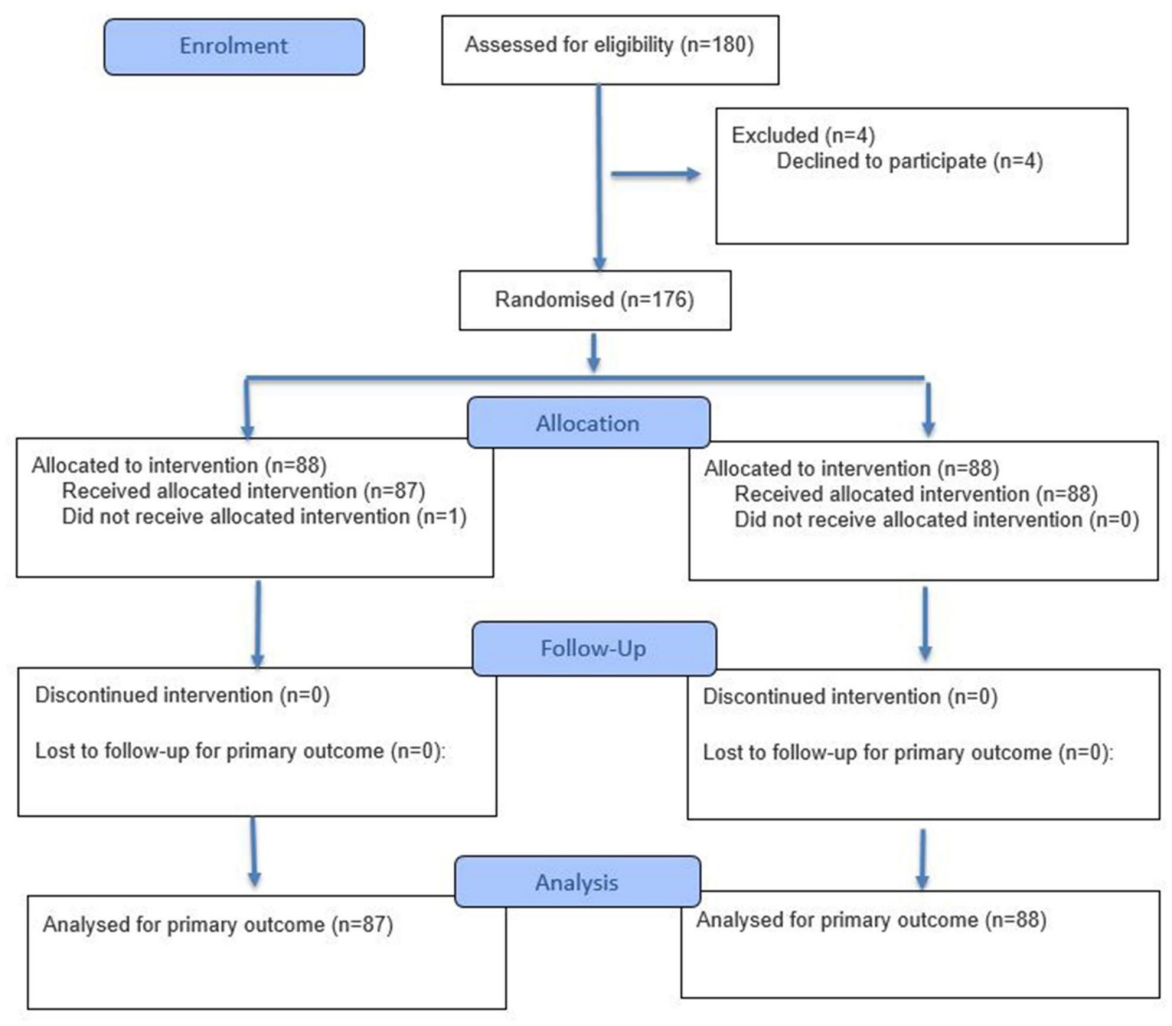
CONSORT 2025 flowchart of patient enrollment, randomization, allocation, follow-up, and analysis

During the study period, all consecutive patients scheduled for elective laparoscopic TEP hernioplasty were assessed for eligibility. A total of 180 patients were screened, and four declined to participate. The remaining 176 patients were randomized (88 to the intervention group and 88 to the control group). One patient in the intervention group did not receive the allocated topical pharmacological application due to an intraoperative protocol deviation and was excluded from the final analysis. Consequently, 175 patients were included in the analysis (87 in the intervention group and 88 in the control group). Patient recruitment, allocation, and analysis are summarized in the CONSORT flow diagram (Fig. 1).

### Sample size

Based on previous studies (Hon et al., 2009) [6] and preliminary analyses, with an effect size of d = 0.516, a significance level of 5% (α = 0.05), and a statistical power of 90%, power analysis indicated that at least 80 patients were required in each group. Considering possible patient losses during the study, the sample size was increased by 10%, and a total of 90 patients were planned for each group. During the study, 3 patients from the intervention group and 2 patients from the control group were excluded. As a result, analyses were performed on data from 87 patients in the intervention group and 88 patients in the control group. The flowchart of the patient selection process is presented in Fig. 1. Sample size calculations were performed using G*Power 3.1 (Heinrich-Heine-Universität Düsseldorf, Germany).

### Randomization and blinding

Randomization was performed using block randomization with blocks of four, with stratification for age (< 50 / ≥50) and sex. The randomization sequence was generated using www.randomizer.org. Allocation concealment was ensured through opaque, sequentially numbered, sealed envelopes prepared by an independent researcher not involved in patient care. On the day of surgery, each envelope was opened by an operating room nurse to assign the patient to the intervention group (receiving topical dexamethasone and bupivacaine application) or the control group. Patient enrollment, group assignment, and outcome assessment were performed by separate researchers.

Due to the nature of the surgical procedure, blinding of the surgical team was not feasible. However, patients were not informed about the exact nature of the local intervention, and postoperative pain assessments were conducted by an independent observer who was blinded to group allocation.

### Surgical technique

All patients underwent laparoscopic TEP hernia repair under general anesthesia. All surgical procedures were performed by one of the authors (G.D.), a board-certified general surgeon experienced in laparoscopic hernia repair. Initially, the preperitoneal space was created through a 10-mm umbilical trocar. Subsequently, two 5-mm trocars were placed along the linea mediana. After careful dissection of the hernia sac, the Cooper’s ligament, spermatic structures, and inferior epigastric vessels were dissected, and the hernia defect was exposed. The defect was covered with a standard 13 × 15 cm Parietene™ macroporous polypropylene mesh (Covidien, Medtronic, Mansfield, MA, USA), placed from the pubic tubercle laterally to the iliopubic tract and the lateral border of the inferior epigastric vessels. The mesh was fixed at three points with an absorbable fixation device (AbsorbaTack™, Covidien/Medtronic): Cooper’s ligament over the pubic tubercle, the inferior border of the rectus muscle, and the lateral anterior abdominal wall.

In the intervention group, after mesh placement, a mixture consisting of 4 mL 0.5% bupivacaine (20 mg) and 2 mL dexamethasone (8 mg), diluted with sterile saline to a total volume of 6 mL, was topically applied to the mesh and surrounding preperitoneal space using a standard laparoscopic irrigation–aspiration cannula, allowing homogeneous distribution without the use of any dedicated spray or aerosolization device. All patients were monitored intraoperatively and postoperatively for potential adverse events, including signs of local anesthetic systemic toxicity, hemodynamic instability, and allergic reactions. In the control group, no topical pharmacological application was performed.

After surgery, all patients received a standardized postoperative analgesia regimen consisting of 1 g intravenous paracetamol every 6 h (maximum 4 g/day). In cases where pain control was insufficient, additional analgesics (50 mg intravenous diclofenac or intravenous tramadol) were administered according to clinical need.

### Pain assessment

Postoperative pain was assessed using the Visual Analog Scale (VAS). Patients were asked to evaluate their pain scores at the 3rd, 6th, and 12th postoperative hours. VAS scores were used to objectively evaluate the severity of postoperative pain. This assessment was performed by a healthcare professional independent of the surgical team. In addition, patient satisfaction was assessed using a five-point Likert scale (1 = not satisfied at all, 5 = very satisfied).

### Primary and secondary outcomes

The primary outcome of the study was postoperative pain intensity, assessed using VAS at the 3rd, 6th, and 12th postoperative hours. Secondary outcomes included additional analgesic requirement, patient satisfaction (5-point Likert scale), postoperative nausea and vomiting (PONV), early complications, surgical time, and length of hospital stay.

### Statistical methods

The data were analyzed using SPSS Version 22.0 (IBM Corp., Armonk, NY, USA) software. Descriptive statistics for categorical variables were presented as numbers (n) and percentages (%). Differences between groups in terms of categorical variables were assessed using the Pearson Chi-square test or Fisher’s exact test, depending on the observed frequencies. Descriptive statistics for continuous variables were reported as mean ± standard deviation (SD) or median (interquartiles: Q1, Q3) according to their distribution. The assumption of normal distribution was assessed statistically using the Kolmogorov–Smirnov test and visually by examining Q-Q plots and histograms. Homogeneity of variances was checked using the Levene test. When normality assumptions were met, the independent samples t-test was used to compare continuous variables between two independent groups; when normality assumptions were not met, the Mann–Whitney U test was used. The Friedman test was used to evaluate the intra-group change in VAS scores measuring pain levels over time, as the parametric assumptions were not met. Where statistically significant differences were detected by the Friedman test, post-hoc analyses involving Bonferroni-corrected pairwise comparisons were performed to determine between which time points the difference occurred. In all statistical tests, the significance level was assessed two-tailed, and *p* < 0.05 was accepted as the statistical significance threshold.

## Results

A total of 175 patients were analyzed, including 87 in the intervention group and 88 in the control group. The mean age of the patients was 42.75 ± 15.47 years (18–69), and the mean length of hospital stay was 1.13 ± 0.37 (1–3) days. Of the patients, 94.9% (*n* = 166) were male and 5.1% (*n* = 9) were female. Additional analgesia was required in 90.3% (*n* = 158) of the patients, and PONV occurred in 9.1% (*n* = 16).

Statistical findings comparing the sociodemographic and clinical characteristics of the study groups are presented in Table 1. Sex distribution was similar in both the intervention and control groups (*p* = 0.496). The mean age values were also similar between the groups (42.91 ± 14.82 vs. 42.6 ± 16.18; *p* = 0.896). The distribution of ASA scores was similar between the groups (*p* = 0.829). Regarding the side of operation, no significant difference was observed between the groups; in the intervention group, 42.5% of cases were operated on the left side and 57.5% on the right, whereas in the control group, 54.5% were operated on the left and 45.5% on the right (*p* = 0.112).

**Table 1:** Statistical findings regarding the comparison of socio-demographic and clinical characteristics between research groups

		Intervention Group (Bupivacaine and Dexamethasone) (*n* = 87)	Control Group (Standard TEP) (*n* = 88)	*P* values
Gender	M	84 (96.6%)	82 (93.2%)	0.496^b^ -
	F	3 (3.4%)	6 (6.8%)	
Age (years)		42.91 ± 14.82	42.6 ± 16.18	0.896^c^ t(173) = 0.130
ASA	I	36 (41.4%)	35 (39.8%)	0.829^c^ 𝜒(1) = 0.047
	II	51 (58.6%)	53 (60.2%)	
Surgical Time		47.4 ± 7.8	46.5 ± 8.1	0.455^c^ t(173) = 0.749
Side	L	37 (42.5%)	48 (54.5%)	0.112^a^ 𝜒(1) = 2.529
	R	50 (57.5%)	40 (45.5%)	
Length of Hospital Stay (days)		1 (1–1) (1.06 ± 0.23)	1 (1–1) (1.2 ± 0.46)	0.011^d^ U = 4.309
Need for Rescue Analgesia	Yes	74 (85.1%)	84 (95.5%)	0.020^a^ 𝜒(1) = 5.392
	No	13 (14.9%)	4 (4.5%)	
PONV	Yes	82 (94.3%)	77 (87.5%)	0.121^a^ 𝜒(1) = 2.402
	No	5 (5.7%)	11 (12.5%)	
Patient Satisfaction Score (Likert Scale)	Neutral	0 (0%)	1 (1.1%)	0.001^b^ -
	Satisfied	27 (31%)	50 (56.8%)	
	Very satisfied	60 (69%)	37 (42%)	

*TEP* Total Extraperitoneal, *M* Male, *F* Female, *L* Left, *R* Right, *PONV* Postoperative Nausea and Vomiting

^a^Chi-square test with n (%)

^b^Fisher exact test with n (%)

^c^Student’s t-test with mean ± SD

^d^Mann Whitney U test with median (Q1, Q3)

There was no statistically significant difference between the groups regarding operative time (*p* = 0.455). The length of hospital stay was higher in the control group, and this difference was statistically significant (1.2 ± 0.46 days vs. 1.06 ± 0.23 days; *p* = 0.011). The need for additional analgesia was higher in the control group (95.5%, *n* = 84) compared to the intervention group (85.1%, *n* = 74), and this difference was statistically significant (*p* = 0.020). Regarding patient satisfaction, 69% of patients in the intervention group reported being “very satisfied”, compared to 42% in the control group; this difference was statistically significant (*p* = 0.001, Table 1).

In the early postoperative period, only minor complications (Clavien–Dindo Grade I–II) were observed. No major complications (Grade III or higher) or mortality (Grade V) were detected in either group. A comprehensive adverse event table summarizing all complications, exact event counts, percentages, Clavien–Dindo severity grades, intervention-relatedness, and their 95% confidence intervals has been added (Table 2). The most common complications in the intervention group were seroma (2.30%; *n* = 2) and scrotal hematoma (2.30%; *n* = 2), both classified as Clavien–Dindo Grade I and considered possibly related to the topical pharmacological application. In the control group, the most common complication was seroma (3.41%; *n* = 3). Wound infection was observed in both groups at Grade II and was assessed as unrelated to the topical pharmacological application. There was no statistically significant difference in the overall incidence of minor complications between the groups (*p* = 0.790). Likewise, no significant difference was found in the frequency of postoperative nausea and vomiting (PONV) between the groups (*p* = 0.121). No cases of hyperglycaemia or delayed wound healing, potential complications associated with steroid use, were detected in any patient. Exact event counts and 95% confidence intervals for each complication are provided in Table 2.

**Table 2:** Postoperative complications by group, severity, and relationship to the intervention

Complication	Intervention Group (*n* = 87)	Control Group (*n* = 88)	Clavien–Dindo Grade	Relationship to Intervention	95% CI (Intervention)	95% CI (Control)
Seroma	2 (2.30%)	3 (3.41%)	Grade I	Possibly related	0.28–8.10	0.71–9.55
Scrotal hematoma	2 (2.30%)	0 (0%)	Grade I	Possibly related	0.28–8.10	—
Wound infection	1 (1.15%)	1 (1.14%)	Grade II	Unrelated	0.03–6.26	0.03–6.25
PONV	4 (4.60%)	7 (7.95%)	Grade I	Unrelated	1.27–11.41	3.27–15.67
Hyperglycaemia (steroid‑specific)	0	0	—	—	—	—
Delayed wound healing (steroid‑specific)	0	0	—	—	—	—

Findings related to intra-group and inter-group comparisons of VAS scores measured at different time points (3, 6, and 12 h) are presented in Table 3. In the intervention group, the median VAS scores were 4 (3rd hour), 3 (6th hour), and 2 (12th hour), with a significant reduction observed in intra-group comparisons (*p* < 0.001). Post-hoc analyses revealed statistically significant differences between all time intervals (*p* = 0.041, *p* < 0.001, *p* < 0.001, respectively). Similarly, in the control group, significant decreases in VAS scores were observed, with median VAS decreasing from 7 to 6 and then to 3 (*p* < 0.001), but the 3rd- and 6th-hour values were markedly higher compared to the intervention group.

**Table 3:** Statistical findings of within-group and between-group comparisons of VAS scores at different time points

Group	VAS at 3rd hour (1)	VAS at 6rd hour (2)	VAS at 12rd hour (3)	*P* values	Post-hoc *P* values
Intervention Group (Dexamethasone and Bupivacaine)	4 (3–4) (3.75 ± 0.58)	3 (3–4) (3.36 ± 0.63)	2 (2–3) (2.34 ± 0.66)	< 0.001^a^ χ²(2) = 105.2 Effect size:0.605	1–2: 0.041 1–3:<0.001 2–3:<0.001
Control Group (Standard TEP)	7 (7–7) (6.88 ± 0.37)	6 (6–7) (6.11 ± 0.79)	3 (3–4) (3.4 ± 0.75)	< 0.001^a^ χ²(2) = 156.1 Effect size:0.887	1–2:<0.001 1–3:<0.001 2–3:<0.001
*P* values	< 0.001^b^ U = 7.650 Effect size: 0.998	< 0.001^b^ U = 7.548 Effect size: 0.972	< 0.001^b^ U = 6.631 Effect size: 0.732		

*TEP* Total Extraperitoneal

^a^Friedman test following post-hoc tests with Bonferroni correction

^b^Mann–Whitney U test; values presented as median (Q1–Q3) with mean ± SD provided additionally

**Fig. 2:**
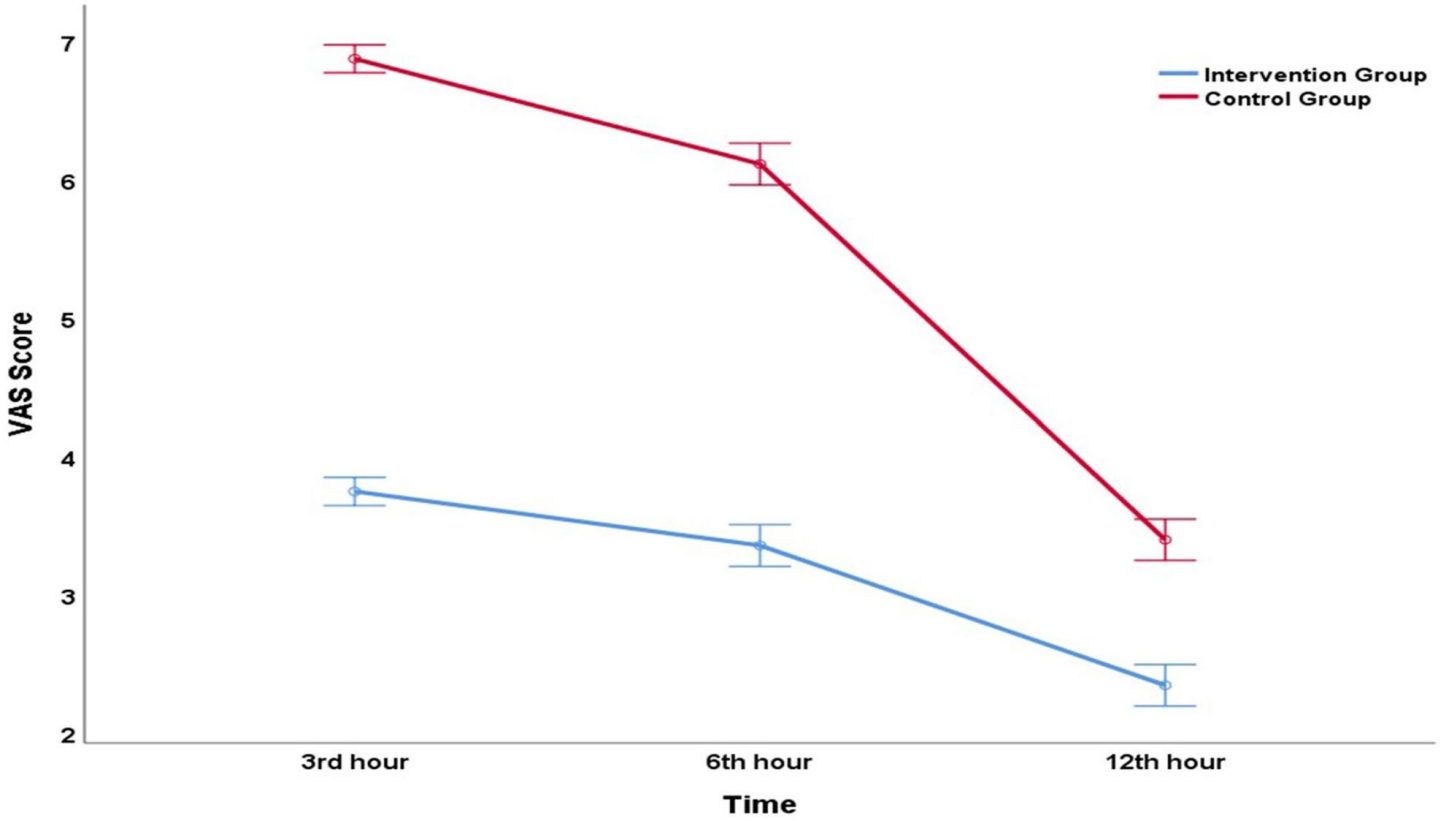
Line graphs illustrating the time-dependent changes in the mean VAS scores and their 95% confidence intervals between the intervention and control groups

In inter-group comparisons, VAS scores differed significantly at each time point: 3rd hour (*p* < 0.001), 6th hour (*p* < 0.001), and 12th hour (*p* < 0.001), with the intervention group reporting lower pain levels (Table 3). Furthermore, differences in VAS scores between the 3rd, 6th, and 12th hours were compared between the groups to examine score changes. The change in VAS scores was significantly greater in the intervention group compared to the control group (3rd–6th hour: *p* = 0.005; 6th–12th hour: *p* < 0.001; 3rd–12th hour: *p* < 0.001). Line graphs showing time-dependent changes in mean VAS scores (in addition to median values) and 95% confidence intervals between the groups are presented in Fig. 2.

## Discussion

The present randomized controlled study demonstrated that topical application of dexamethasone combined with bupivacaine to the preperitoneal space during TEP hernioplasty significantly reduced early postoperative pain, decreased the need for additional analgesics, and improved patient satisfaction. Although the use of local anesthetics in the preperitoneal space has been previously investigated, the duration and clinical relevance of their analgesic effect remain controversial. Our findings suggest that the addition of dexamethasone may enhance and prolong the analgesic efficacy of bupivacaine in the early postoperative period.

Previous studies evaluating bupivacaine alone after TEP repair have reported a reduction in early postoperative pain; however, this effect has generally been described as short-lived. O’Riordain et al. observed lower pain scores at discharge but no difference beyond the early postoperative phase [5], while Hon et al. demonstrated effective pain control up to 24 h with preemptive bupivacaine infiltration, with diminishing effects thereafter [6]. In contrast, our study showed sustained analgesic benefit up to 12 h, accompanied by reduced rescue analgesic use, suggesting that the anti-inflammatory properties of dexamethasone may contribute to prolonging the clinical effect of local anesthesia.

Postoperative pain following TEP surgery has also been attributed to diffuse pain originating from the preperitoneal dissection area, described as “dissectalgia” by Kumar et al. [8]. Their findings highlighted the importance of direct pharmacological intervention at the dissection site to reduce this regional pain component. Consistent with this concept, our results indicate that local application of a bupivacaine–dexamethasone combination to the preperitoneal space effectively attenuates early postoperative pain and reduces analgesic requirements, supporting the benefit of targeting the primary source of nociception.

Several randomized trials and meta-analyses have suggested that local anesthetics alone may have limited or inconsistent efficacy following TEP hernioplasty. Studies by Çolak et al., Subwongcharoen and Udompornmongkol, Abbas et al., and Lau et al. reported that while bupivacaine reduced pain in the early hours, this effect was not sustained and was often comparable to placebo beyond the first postoperative day [7, 9, 16, 17]. Similarly, a meta-analysis by Tong et al. concluded that extraperitoneal bupivacaine did not provide significant superiority over placebo [18]. In contrast, the consistent reductions in pain scores and analgesic consumption observed in our study emphasize the clinical advantage of combining local anesthetics with an anti-inflammatory agent rather than relying on local anesthesia alone.

Beyond its analgesic properties, dexamethasone exerts a potent local anti-inflammatory effect that may influence mesh–tissue interaction following TEP hernioplasty. Excessive postoperative inflammation in the preperitoneal space has been associated with increased fibroblast activity, exaggerated collagen deposition, seroma formation, and mesh-related adhesions, potentially contributing to chronic pain and impaired mesh incorporation [11, 19, 20]. By suppressing pro-inflammatory cytokines, reducing capillary permeability, and limiting early exudative responses, topical dexamethasone may modulate—rather than inhibit—the inflammatory cascade, thereby creating a more favorable biological environment for controlled mesh integration [10, 11]. This pharmacological effect is particularly relevant in TEP hernioplasty, where postoperative pain predominantly originates from the preperitoneal dissection plane rather than skin incisions. Local application of dexamethasone allows targeted modulation of inflammation within this confined anatomical space, while minimizing systemic exposure. Importantly, no increase in mesh-related complications, including seroma, infection, or delayed wound healing, was observed in the intervention group, supporting the safety of this approach.

Recent evidence further supports the concept of local steroid efficacy in laparoscopic surgery; Bauiomy et al. demonstrated that intraperitoneal dexamethasone, alone or in combination with dexmedetomidine, significantly reduced postoperative nausea and vomiting following laparoscopic cholecystectomy, reinforcing the biological plausibility of locally administered steroids in modulating postoperative inflammatory and nociceptive responses [21].

The clinical relevance of these findings is further supported by their alignment with Enhanced Recovery After Surgery (ERAS) principles, which advocate multimodal, opioid-sparing analgesia to optimize postoperative recovery [13, 19, 20]. In our study, effective pain control was achieved using paracetamol-based analgesia without routine opioid requirement, and patient satisfaction was significantly higher in the intervention group. The synergistic interaction between bupivacaine and dexamethasone—through sodium-channel blockade and suppression of inflammatory mediators, respectively—provides a plausible mechanistic explanation for the sustained analgesic effect observed [5, 16, 17]. Although long-term assessment of mesh incorporation was beyond the scope of this study, our results suggest that topical dexamethasone combined with bupivacaine represents a simple, safe, and cost-effective adjunct for improving early postoperative outcomes following TEP hernioplasty.

### Strengths and limitations

This study has several methodological strengths. Its prospective, randomized controlled design and sample size determined by power analysis enhance internal validity, while age- and sex-matched randomization helped minimize potential confounding. In addition to postoperative pain scores, clinically relevant outcomes such as rescue analgesic requirements and patient satisfaction were evaluated, allowing a more comprehensive assessment of postoperative analgesia. The topical application technique used in this study was simple, low-cost, and easily applicable in routine practice, and the evaluation of a combined local anesthetic–steroid approach in the preperitoneal space adds originality to the existing literature.

Several limitations should also be acknowledged. First, postoperative pain was assessed using the Visual Analog Scale, a subjective measure that may be influenced by individual pain perception despite its widespread acceptance [22, 23]. Second, pain assessment was limited to the first 12 postoperative hours. This period was selected to capture peak pain intensity and the expected pharmacological effects of bupivacaine and dexamethasone; however, this design does not allow conclusions regarding the duration of analgesic benefit beyond the early postoperative phase.

Third, the study population consisted only of patients undergoing elective unilateral TEP hernioplasty at a single center, which may limit the generalizability of the findings to more complex cases or other laparoscopic procedures. Operator-related factors, including surgeon and team experience, also cannot be fully excluded. In addition, the absence of direct radiological or histopathological assessment of mesh incorporation represents a limitation of this study; therefore, conclusions regarding the long-term effects of dexamethasone on mesh integration should be interpreted cautiously.

Another limitation is that partial systemic absorption of dexamethasone after local application cannot be entirely ruled out, making it difficult to distinguish purely local effects from potential systemic contributions. Moreover, the lack of an equivalent-volume placebo application in the control group may limit the ability to exclude a mechanical effect related to fluid instillation.

Finally, the absence of full blinding and the lack of a placebo application represent methodological limitations that may introduce bias. In particular, the lack of blinding of both patients and surgeons may have resulted in performance bias, especially for subjective outcomes such as pain perception and patient satisfaction. Moreover, the absence of an equivalent-volume placebo application in the control group means that a potential placebo effect related to fluid instillation or patient expectation cannot be fully excluded. The retrospective registration of the study on ClinicalTrials.gov also represents a methodological limitation and should be considered when interpreting the findings. Analgesic consumption was recorded based on the need for additional analgesia rather than objective cumulative dosing, and the short follow-up period means that statements regarding the absence of systemic side effects apply only to the early postoperative period. Therefore, longer-term, multicenter randomized studies incorporating placebo-controlled designs and extended follow-up are warranted.

## Conclusion

This prospective randomized controlled study demonstrated that the combination of dexamethasone and bupivacaine applied to the surgical site during TEP hernioplasty significantly reduced postoperative pain scores and decreased the need for additional analgesics in the early postoperative period. Patients in the intervention group reported significantly lower VAS scores at the 3rd, 6th, and 12th hours compared to the control group and achieved higher levels of patient satisfaction. Moreover, this topical pharmacological application did not prolong surgical time and did not cause any additional complications. The findings indicate that the local administration of dexamethasone and bupivacaine provides an effective and safe method of pain control in TEP surgeries. Its simplicity, low cost, and absence of systemic side-effect risk make this method a practical option in daily surgical practice. However, multicenter and long-term randomized studies comparing different combinations and application timings are required to more robustly demonstrate the long-term efficacy and generalizability of this approach.

## Data Availability

Available on request from the corresponding author.

## Abbreviations

ASA: American Society of Anaesthesiologists
PONV: Postoperative Nausea and Vomiting
SD: Standard Deviation
SPSS: Statistical Package for the Social Sciences
TEP: Total Extraperitoneal
VAS: Visual Analog Scale
